# Co-Occurrence of Violence on the Severity of Abuse in Intimate Relationships

**DOI:** 10.1177/08862605211064238

**Published:** 2021-12-30

**Authors:** Frédéric Ouellet, Emeline Hetroy, Guisela Patard, Christophe Gauthier-Davies, Chloé Leclerc

**Affiliations:** 1 School of Criminology, 5622 Université de Montréal, Montreal, QC, Canada; 2 Psychology, 5622 Université de Montréal, Montreal, QC, Canada; 3 6775 ENAP, École nationale d’administration publique, Quebec, QC, Canada

**Keywords:** intimate partner violence (IPV), severity, physical violence, sexual violence, co-occurrence of violence, violence against women, life history calendar

## Abstract

Several empirical studies have shown that women who experience violence in intimate personal relationships (IPV) commonly experience more than one form of violence. While it is recognized that individual trajectories of IPV vary over time, little is known about the temporal dynamics of this co-occurrence or its consequences. This study describes the different forms of violence experienced by women and looks at whether it is possible to predict when severe violence (physical and sexual) is most likely to occur. Data in the study comes from interviews with 70 women who had been victims of intimate partner violence. The life history calendar method was used to facilitate identifying kinds and levels of violence and the month in which violence took place. Individual victimization trajectories were found to be heterogenous and likely to change in the short term. The women in our sample experienced more than one form of intimate partner violence and co-occurrence of different forms of violence was common in individual trajectories. The characteristics of the kind of violence experienced were important in understanding the temporal aspects of acts of severe violence. The increased knowledge about patterns of violence provided by these results should help to develop better ways to intervene to prevent such events.

Intimate partner violence (IPV) is an important health issue (Sonis & Langer, 2008). Women who experience IPV have poorer health than women who are not abused (Bonomi et al., 2006) and the mental health of women who experience the co-occurrence of severe physical, emotional, and sexual abuse is worse than that of women in similar contexts who experience other forms or levels of abuse (Hegarty et al., 2013). Several empirical studies have found that women who experience IPV are likely to experience more than one form of violence (McFarlane et al., 2005; Patard et al., 2020; Winstok, 2008). While it is known that IPV varies over time in individual trajectories, little is known about the temporal dynamics behind these changes or the consequences associated with them. Do repeated acts of violence have an aggravating effect on the level of violence suffered? Does the presence of some kinds of violence make it possible to predict escalation in the level of violence?

Finding a way to identify when IPV is likely to escalate is important, as some studies have shown that violence can increase until it results in death (Campbell et al., 2007; Zara & Gino, 2018). Little is known about the factors and circumstances associated with such escalation and very few empirical studies have looked at violent acts in intimate relationships as part of a sequence of events that vary in presence, frequency, and severity.

Although IPV is not a rare phenomenon (Johnson, 2008), knowledge about the developmental aspects of this violence—how it unfolds over time—is fragmentary. Previous explorations of the dynamics of IPV conflicts have relied primarily on qualitative methods (Winstok, 2008) so our research project provides a new perspective on the issue. Using the life history calendar method (LHC), we reconstructed the sequence of IPV and associated events for each victim, making it possible to study violence in particular contexts and trajectories. Our aim was to describe the different forms of violence suffered by women to determine if it is possible to predict when changes in levels and frequency of physical and sexual violence are likely to occur. It is hoped that understanding various types of violence and the links between them will lead to a better understanding of patterns of violence and help in developing methods for intervention and prevention.

## Dynamics of Intimate Partner Violence

While there are many studies of IPV, most of them focus on a specific event, creating a static portrait that fails to recognize that each episode of violence is part of a sequence of events, a trajectory that varies depending on context and time. In contrast, several theoretical models have attempted to explain both violence and its repetition. Walker (1984) used the term “cycle of violence” to capture the temporal dimension of the phenomenon and the sequence of events it may involve, describing IPV as a predictable sequence of behaviors in which the calm phases (honeymoon/reconciliation phases) become shorter and shorter while the crisis phases increase in intensity. However, this model has little empirical validity, does not reflect the full experiences of women who are victims of domestic violence, and suggests that violence demonstrates a chronic and ascending evolution while more recent work tends to show that violence can also be isolated, stable, or decrease (e.g., Piquero et al., 2006; Winstok, 2008). Johnson (1995) notes that escalation of violence is often associated with the trajectories of victims of intimate terrorism or coercive controlling violence. In this type of situation, the motivation for violence is control and power. The perpetrator, usually male, uses control tactics, both violent and non-violent, to create a climate of terror in which the victim loses both will and ability to resist. Violence is then more frequent and severe, reflecting the intent to dominate and the passivity of the victim. Comparative analysis of clinical and population data suggests that the prevalence rate for intimate terrorism in the general population is between 2% and 4% (Jonhson, 2008). The individual paths of victimization in IPV appear to be heterogenous and more in-depth study of the factors likely to influence them is still needed.

Knowledge about the developmental dynamics of IPV is limited (Halpern et al., 2009), although both official (Ouellet, Blondin, Leclerc & Boivin, 2017) and self-reported data (Hayes, 2016; Kerr, Whyte & Strang, 2017; Piquero et al., 2006) suggest that IPV is often repeated over time and some researchers have proposed that it be conceptualized dynamically (Capaldi & Kim, 2007) to facilitate consideration of both the non-linear aspects of the phenomenon (Katerndahl et al., 2014) and its developmental course. Most research on the dynamics of IPV has relied on cross-sectional surveys or qualitative interviews, meaning that little quantitative data is available. However, despite the scarcity of data, some studies have attempted to identify different patterns of violence in an intimate partner context. Dutton and colleagues (2005) used cluster analysis on information from a sample of 406 women seeking assistance in dealing with IPV perpetrated by a current or former male partner and identified three patterns based on the intensity of the physical, psychological, and sexual violence suffered. The characteristics of the women associated with each of the patterns differed in several aspects (e.g., employability, PTSD and depression, and quality of life). In a study of 200 adult women in violent relationships, Katerndahl and his colleagues (2014) looked at whether the dynamics of IPV contributed to different outcomes. Their analysis identified three dynamic patterns of violence (periodic, chaotic, and random), which were correlated with outcomes that varied from developing ways to cope to requiring medical treatment. Using the life history calendar method, Patard et al. (2020) examined the victimization journey of 75 women who had experienced at least one form of intimate partner violence within a 3 year window. They found that different forms of violence (psychological, economic, physical, and sexual) followed three temporal patterns (episodic, intermittent, and continuous). Although the conceptual definitions of different pathways vary in these three studies, they all conclude that violence evolves differently in individual trajectories. Researchers have suggested that it is difficult to predict patterns because in many cases the violence suffered is unique to a particular situation (Dutton et al., 2005; Katerndahl et al., 2010) and may have been influenced by multiple factors (e.g., individual characteristics of the protagonists, relationship characteristics, and life circumstances). These previous studies suggest the importance of understanding the elements likely to be associated with changes in the trajectory of violence in order to evaluate the contexts in which violence occurs.

Studies on variations in violence over time can be divided according to the parameters of the violence examined: some are interested in the presence/frequency of IPV, while others try to explain how levels (severity) of such violence change over time. In their study on the long-term risks of being physically assaulted in an intimate relationship based on a sample of 87 women of Philippine descent, Yoshihama et al. (2011) found that physical IPV increased during the early period of the relationship and then declined. Hayes' (2016) study of 497 victims of IPV highlights the effect of employment and length of separation. While employment of both perpetrator and victim and of only the victim decreased the risk for and frequency of violence, employment of only the abuser and length of separation decreased only frequency of physical abuse. Blondin, Ouellet, and Leclerc (2018) looked at retrospective data from 53 women who experienced IPV over a period of 36 months and found that being in a relationship (as opposed to being separated or being in touch with an ex-partner) and cohabitation increased the frequency of physical violence during the time considered. They also show that in months in which psychological violence is more intense, episodes of physical violence also tend to occur more frequently. These studies suggest that it is possible to predict the parameters of IPV and to identify the various factors associated with changes.

Ouellet et al. (2016) attempted to predict changes in the severity of IPV incidents that involved current or former partners and had been reported to the police in a large metropolitan city in Canada (*N* = 53,429 incidents) between 2000 and 2009. Their results show that each new incident involving the same aggressor or victim increases the likelihood that future incidents of IPV will be more serious. The study also found that incidents of IPV involving aggressors or victims who have a criminal record for offenses other than IPV are likely to be more serious. Barnham and colleagues (2017) looked at unique perpetrator–victim couples involved in 140,998 IPV incidents reported to Thames Valley Police (England) between 2010 and 2015 and found no evidence of an increase in the seriousness of acts committed during a 731 day observation period. They conclude that a small proportion of aggressors are responsible for most of the reports of incidences of serious harm. Another study carried out in Australia reached similar results: looking at police records on incidents of IPV involving unique couples over a 5 year period (*N* = 61,796), Kerr et al. (2017) found only a few examples of escalation in the severity of IPV over the period studied while considering only couples with two or more incidents during this period revealed a strong pattern of escalation in the seriousness of offending—sometimes up to 20 incidents over 4 years. However, these results must be interpreted with caution as police data often has a significant selection bias, particularly in cases of IPV, which are likely to be underreported to the police (Wolf et al., 2003). Piquero et al. (2006) analyzed self-reports from the Spouse Assault Replication Program and found that the severity of physical assaults occurring in a marital context increased, decreased, or remained stable over the 24 months observed. They conclude that individual victimization trajectories are heterogenous and likely to change in the short term (Piquero et al., 2006). These studies tend to agree that the number of incidents of IPV vary between couples but there was no consensus about how changes in frequency or in the seriousness of such incidents can be explained.

Only a few researchers have tried to provide explanations of why IPV varies over time. Yakubovich and colleagues (2018) conducted a meta-analysis of previous longitudinal research on risk and protective factors for intimate partner violence and found that research often focused only on physical violence, with little attention to other types of concurrent violence. Some researchers have, however, looked at violence other than physical. Winstok (2008) reports that physical violence in intimate relationships is often accompanied by other forms of violence and several studies, based on data from both victims or perpetrators of violence, have found co-occurrence of psychological, physical, and sexual victimization involving women victims (Basile & Hall, 2011; Hamby & Grych, 2012; Patard et al., 2020; Sullivan et al., 2012). Basile and Hall (2011) studied men who had been arrested for physical assault on a partner and found that 96.8% of their victims reported having experienced psychological, physical, and sexual violence as well as criminal harassment. Sullivan et al. (2012), in a study of women victims of partner violence, found that psychological violence was the most frequent form of violence and that severe psychological violence was almost systematically accompanied by physical and sexual violence. A study by Patard et al. (2020) found that 32% of their sample had experienced economic, psychological, physical, and sexual violence, and 40% had experienced three of these. While some authors have documented the extent of the co-occurrence of different manifestations of psychological, physical, and sexual violence (Humphreys & Thiara, 2003; Logan et al., 2007), discussions of the context in which they occurred and their variations over time were limited.

Some studies have focused on associations between different forms of violence over time. Frye and Karney (2006) considered within-subject variability in aggressive behavior in a sample of newlywed couples and found that spouses were more likely to engage in physical aggression during periods of higher levels of psychological aggression. Blondin et al. (2018) also found a temporal association between intense psychological violence and frequency of physical aggression. However, with a few exceptions, most prior research has not considered the spectrum of violence that women experience in intimate relationships and the consequences of the co-occurrence of such violence (Hamby & Grych, 2012).

## Current Study

Studies that recognize the relevance of examining transitions in IPV over time have demonstrated that women experience unique patterns and kinds of abusive acts in the context of intimate relationships. Understanding how violence evolves therefore requires consideration of the characteristics of the victim, the partner, and their relationship as well as their life circumstances. Some previous studies have recognized that co-occurrence of various levels and kinds of IPV is the norm, not the exception (Hamby & Grych, 2012). However, while these studies point to the need to consider co-occurrence of violence in individual paths, they provide little information about individual trajectories or the consequences associated with them. Yakubovich and his collaborators (2018) note that only a few studies use data pertaining to several types of violence, with most studies exploring various types of behaviors related to a single kind of violence (Hall et al., 2012), making it difficult to understand the distinctions and similarities between the different types of violence. Correcting these omissions requires more complete consideration of the entire constellation of violence experienced as well as longitudinal assessment of relationships between patterns of violence and outcomes.

The present research focuses on individual trajectories of victimization and the temporal dynamics that accompany serious violence in the context of intimate relationships. The characteristics of protagonists, their relationship, and their life circumstances are considered, as is the effect of various forms of violence on the ability to predict the occurrence of serious physical and sexual violence. The various manifestations of violence during the period under study are detailed and a method for predicting the occurrence of serious physical and sexual violence over time is proposed.

## Method

The data used in this study was taken from structured interviews conducted between 2014 and 2016 with 70 women who had been victims of intimate partner violence. This project received the approval of the research ethics committee of the University of Montreal, all the women involved agreed to participate in this study and gave free and informed consent. They were assured of the confidentiality of the data and received financial compensation for their participation. Participants had been referred by several agencies located in geographically diverse regions of the province of Quebec, Canada. These institutions and organizations include shelters for women victims of violence and their children (*n* = 36), victim assistance services (*n* = 9), correctional services (*n* = 11), community organizations (*n* = 2), and therapy center (*n* = 3). Women were also recruited through public notices and ads in social media (*n*=9). The plurality of recruitment sites allowed for more diversification in the pathways under study. Eligibility criteria were 1) female, 2) aged 18 and over, and 3) victim of at least one form of domestic violence (economic, psychological, physical, or sexual) in the last 3 years. Average length of interview was two and a half hours. The sample is made up mainly of women whose mother tongue is French (97.1%) and who consider themselves to be of Canadian origin (85.7%).

Face-to-face interviews with participants were chosen as the preferred approach as they increase the reliability of answers, especially for more complex questions associated with the timing of life events and violence in the context of an intimate relationship. All the interviews were conducted in French. The survey collected information on areas that were likely to affect the kind and level of violence suffered by participants, such as the characteristics of the relationship and of the two protagonists as well as life circumstances. Information gathered related mainly to the 36-month window before the month in which the interview was conducted.

The life history calendar (LHC) method was used to facilitate gathering information on violence suffered and its monthly context. Developed by Freedman et al. (1988), the LHC is a retrospective research framework for recording events in developmental trajectories that makes it possible to collect detailed information on individual-level event timing and sequencing. It was first used in criminology in the study of criminal careers, where it has proved to be reliable (Hayes, 2018; Sutton et al., 2011). The LHC has been found to improve the quality of retrospective data not only in terms of event synchronization but also by capturing a more accurate report of the sequence of events and changes in individual trajectories (Freedman et al., 1988). In the present study, the themes covered were selected for their theoretical or empirical relevance. The order in which themes were discussed in the questionnaire was determined based on difficulty of recall, beginning with events easier to remember and gradually continuing to events that were more difficult to recall. This order is adapted to the structure of autobiographical memory and facilitates recall through techniques that rely on the sequential and hierarchical storage of memory (Belli, 1998)^1^.

### Data Structure and Risk Exposure

The data used in this study are nested, with the months that make up the window period nested within the relationships that women have maintained during this period. Information was collected on 2520 months of observation (70 victims x 36 months). Most participants (80%) had been involved in a single intimate relationship during the 36 month period, while 20% had had two relationships, making it possible to examine the characteristics of the occurrence of serious physical and sexual violence in 84 intimate relationships. To be at risk, the victim must have been 1) involved in an intimate relationship (*n* = 1931 months) or 2) maintaining contact with a former partner (*n* = 230 months)^2^. Months without contact with a partner or ex-partner were excluded from the analysis (*n* = 412 months). The analyses thus relate to a sub-sample of months during the window period (on average 30.1 months per participant). Two levels of factors were considered. The first level includes factors that can vary over time on a monthly basis, while the second level considers the characteristics of participants, partners, and relationships. The descriptive statistics of the factors (levels 1 and 2) are presented in Table 1.

**Table 1.** Descriptive Statistics of Dynamic and Static Factors.

	Mean (SD)/*N* (%)
Level 1: Dynamic Characteristics (*n* = 2108)	
**Severe physical violence** (1 = yes; 0 = no)	416 (19.7%)
**Severe sexual violence** (1 = yes; 0 = no)	162 (7.7%)
Emotional limitations (1 = yes; 0 = no)	378 (17.9%)
Physical limitations (1 = yes; 0 = no)	200 (9.5%)
Employment (1 = yes; 0 = no)	997 (47.3%)
In relationship (1 = yes; 0 = no)	1931 (91.6%)
Cohabitating (1 = yes; 0 = no)	1246 (59.1%)
Intense psychological violence (1 = yes; 0 = no)	363 (17.2%)
Economic violence (1 = yes; 0 = no)	874 (41.5%)
Sexual violence (1 = yes; 0 = no)	318 (15.1%)
Physical violence (1 = yes; 0 = no)	654 (31.0%)
Nb of previous months with physical violence (number of months)	3.4 (5.5)
Nb of previous months with sexual violence (number of months)	1.8 (6.8)
**Level 2: Static characteristics (*n* = 84)**	
Age	32.8 (10.0)
Education high school not completed	27 (32.1%)
High school completed	11 (13.1%)
Collegial or vocational school	31 (36.9%)
University	15 (17.9%)
Children (1 = yes; 0 = no)	57 (67.9%)
Criminal record (1 = yes; 0 = no)	24 (28.6%)
Partner - age	38.6 (12.2)
Partner - daily alcohol use (1 = yes; 0 = no)	29 (34.5%)
Partner - hard drug use (1 = yes; 0 = no)	43 (51.2%)
Partner - criminal record (1 = yes; 0 = no)	50 (59.5%)

### Dynamic Factors

#### Severe physical and sexual violence

Physical and sexual violence in intimate relationships were measured using the revised version of the Conflict Tactics Scales (CTS2) developed by Straus et al. (1996). To allow examination of monthly changes, participants were asked to identify, for each item, the frequency of the behavior during each month of the window period. Measuring severe physical and sexual violence follows the guidelines proposed by Straus and colleagues (1996). Severe physical violence was measured based on responses to seven items^3^ and serious sexual violence on responses to four items^4^. The study was aimed at determining whether it is possible to predict the occurrence (yes or no) of severe violence for each month during the window period. Severe physical violence was reported for 19.7% of the months in the window period and had been experienced by the majority (70%) of women in our sample. Among women who had suffered episodes of severe physical violence, this violence took place, on average, in 6 months of the window period. Incidences of sexual violence affected 24.3% of women and occurred during 7.7% of the months being considered. When this severe violence occurred, it took place in 2.3 months of the window period.

#### Violence in Intimate Relationships During the Window Period

To measure changes in psychological violence over time, participants were first asked to report on the presence or absence of psychological violence using the eight items from the revised version of the Conflict Tactics Scales (CTS2) for each month of the window period and were then asked to identify whether, according to them, the perceived level of violence was low, average, or high during each month in which it occurred. The measure for psychological violence used in the models is based on intense (high) psychological violence. With economic violence, participants were asked if they had suffered economic violence during the window period using the scale developed by Graham-Kevan and Archer (2003; scale of economic control) and those who answered yes were asked to identify the months in which they had experienced such violence.

Using the seven items from the revised version of the Conflict Tactics Scales (CTS2), the occurrence of sexual violence (yes or no) was determined for each month of the window period. The models that examine changes in severe physical violence over time also capture whether sexual violence occurred in the same month. The models also attempt to capture the cumulative effect of physical violence to determine if the risk of serious physical violence is greater when this form of violence occurs during a greater number of months in the window period or over a consecutive period of several months. The same logic was applied to models concerning serious sexual violence as related to monthly occurrence of physical violence (a 12-item measure based on the CTS2 Physical Assault Scale) and the cumulative effect of sexual violence*.*

#### Life Circumstances

This category looked at the monthly occurrence of factors that vary over time. Using the life history calendar, victims were asked to indicate the months in which certain circumstances were present: experiencing emotional and/or physical limitations, employed, in a relationship, and cohabitating. These variables are coded dichotomously, (0 = absent, 1 = present).

Emotional or physical limitation was identified based on the presence of a physical, psychological, emotional, or mental condition that limited activities at home, at work, at school, or in any other way. The scale used was based on the questions on health status and activity limitations in Statistics Canada’s General Social Survey. Participants were asked to use the life history calendars to indicate the frequency of such limitations and identify the months in which they had occurred. Emotional limitations affected 32 participants in 18% of the months during the period under consideration. Physical limitations were less frequent, appearing in the trajectory of 10 women and in 9% of the months examined.

Periods of unemployment may influence the frequency and severity of occurrences of violence (Hayes, 2016). In our sample, 68.6% of the participants were employed at some point during the observation period and employment lasted an average of 20.8 months.

Most of the participants had experienced separation (temporary or permanent) or divorce during the period under examination but 34.3% maintained contact with a former partner for a variety of reasons (e.g., child custody and divorce proceedings). To measure the effect of separation, a dichotomous variable was created reflecting months in relationship and months of contact with an ex-partner. Months in intimate partner relationships account for 92% of the months observed; episodes of contact with a former partner occurred, on average, during 8 months.

Cohabitation during the period under study was reported by 85.7% of the participants, with periods of cohabitation generally a little less than 2 years (20.8 months on average).

### Static Factors

#### Victim and Partner Characteristics

Information on victim and partner characteristics were provided by participants. Participants were, on average, 33 years old at the beginning of the window period. One-third (33%) had not completed high school, 13% had a high-school diploma, 37% had a vocational school/college diploma, and 18% had a university degree. As the presence of children can modulate the level of violence their presence or absence was considered, with 68% of women reporting one or more child living with them. Recent studies have shown the impact of criminal history on the severity of IPV (Ouellet et al., 2016, 2017), with victims of IPV with criminal records 17% more likely to be involved in more serious incidents of violence (Ouellet et al., 2016). A total of 29% of the women interviewed reported having a criminal record.

Empirical studies of partners or former partners involved in IPV have identified certain characteristics. In our sample, the average age of partners was 39 years old, which is older than the average age of victims (33 years). Victims were asked to quantify their partner’s average alcohol and drug use during the 36 month window period. One-third of partners (35%) were reported to use alcohol daily and more than half (51%) used drugs during the window period. More than half (60%) of partners had a history of crimes other than IPV.

### Analytic Strategy

Generalized linear mixed (GLM) models were used to predict severe physical and sexual violence in the trajectories of female victims of intimate partner violence. HLM version 6.06 (Scientific Software International, Inc., Skokie, IL) was used in this study. GLM models are used to identify the antecedent (static) and intervening (dynamic) factors that have a direct effect on the probability of serious physical and sexual violence and are based on a logic similar to logistic regression. Data obtained at regular intervals is structured hierarchically: months at risk of IPV are nested within individual paths. This type of analysis is distinguished by its flexibility, which makes it useful for this study, as the number of months observed varied between individuals and understanding the context of IPV in relation to the risk of IPV requires analysis of specific sequences in an individual’s trajectory. Our models help understand the role of violence in individual trajectories as well as the effect of individual characteristics and life circumstances and make it possible to examine intraindividual changes in life circumstances as well as differences between victims.

## Results

To better understand violence in intimate relationships, we identified all incidences of violence in the context of an intimate relationship during the window period, dividing theses incidences according to frequency and type. This made it possible to contextualize such violence and allowed further examination of the dynamics underlying severe violence.

### Violence in Intimate Relationships During the Window Period

While the focus of this study is on severe physical and sexual violence, such violence cannot be understood without considering other forms of violence. To create a more complete picture of IPV during the period under examination we looked at prevalence (the proportion of women who experienced at least one episode of one kind of IPV during the window period) and frequency (the number of times the violence occurred) for each form of violence. Almost all women (92.9%) in the sample reported having experienced psychological violence, with physical violence (73.8%) the second most prevalent form of violence. Although the prevalence of economic (54.8%) and sexual violence (38.1%) was lower, these forms of violence were still experienced by a significant proportion of women.

We then looked at whether such victimization occurred regularly or as isolated events, focusing exclusively on months in which the women were in intimate relationships or in contact with an ex-partner. During the months in which women were at risk, psychological abuse occurred in 71.6% of the months, economic violence in 41.5%, and physical violence in 31.0%. Sexual violence occurred in 15.1% of the risk period. These descriptive results suggest that the violence experienced by participants was regular rather than episodic.

Co-occurrence of types of violence during the window period was also examined. In the 84 relationships considered, no violence was recorded in 4.8% of relationships, one form in 11.8%, two forms in 31.0%, three forms in 34.5%, and four forms in 17.9%, indicating that most of these relationships were marked by violent behaviors and that most of the women in the study experienced more than one form of violence.

There did not seem to be any dominant pattern in frequency of occurrence of forms of violence during the window period (Table 2). There was no violence in almost 1 month out of five, almost half (49.6%) the months were marked by a single form of violence, and multiple forms of violence occurred in 32.1% of the months.

**Table 2.** Descriptive Statistics of the Co-Occurrence Of Violence During the Window Period.

*N* = 2108	No. of months	% of months
(0) No violence	417	19.8
(1) Only psychological violence (average or below average)	414	19.6
(1) Only intense psychological violence	88	4.2
(1) Only economic violence	331	15.7
(1) Only physical violence	151	7.2
(1) Only sexual violence	69	2.9
(2) Intense psychological violence and economic violence	56	2.7
(2) Intense psychological violence and physical violence	75	3.6
(2) Intense psychological violence and sexual violence	10	0.5
(2) Economic violence and physical violence	178	8.4
(2) Economic violence and sexual violence	68	3.2
(2) Physical violence and sexual violence	10	0.5
(3) Intense psychological violence, economic violence, and physical violence	80	3.8
(3) Intense psychological violence, economic violence, and sexual violence	9	0.4
(3) Economic violence, physical violence, and sexual violence	114	5.4
(4) Intense psychological violence, economic violence, physical violence, and sexual violence	38	1.8

Our descriptive statistics show the prevalence of each form of intimate partner violence, demonstrating that co-occurrence of violence was very likely within individual trajectories. We then looked at possible associations over time between occurrences of forms of violence and severe physical or sexual violence.

### Predicting Severe Physical and Sexual Violence

To predict occurrences of severe physical and sexual violence over time, we looked at individual characteristics as well as the life circumstances associated with different forms of violence.

#### Severe Physical Violence

Our findings indicate that, in general, level of education was related to the level of risk of severe physical violence during the window period, with those with more education at lower risk. The results also suggest that age of the partner matters: the younger the partner, the higher the risk of serious physical violence. Monthly variations in life circumstances or certain temporary limitations in daily life had little impact on the occurrence of severe physical violence and the significant effect of cohabitation faded when other dynamic characteristics were introduced (Table 3 - Model 2). However, occurrence of other forms of violence significantly predicted months of severe physical violence. More specifically, risk of serious physical abuse was increased during months when more intense psychological violence was reported (2.0 times more at risk) and in months in which the victim reported having been the target of economic violence (3.4 times more at risk). The risk of serious physical violence increases by 14% for each additional month that women are victims of physical violence (regardless of the level of severity). This suggests an aggravating effect for physical violence over time.

**Table 3.** Multilevel Logistic Regression Predicting Severe Physical Violence.

Severe Physical Violence	Model 1: Life Circumstances		Model 2: Violence Suffered	
	γ (S.E.)	OR [CI]	γ (S.E.)	OR [CI]
Level 1: Dynamic characteristics (*n* = 2108)				
Emotional limitations	0.22 (0.33)	n.s	−0.13 (0.37)	n.s
Physical limitations	0.55 (0.37)	n.s	−0.04 (0.23)	n.s
Employment	0.04 (0.26)	n.s	−0.11 (0.28)	n.s
In relationship	−0.02 (0.21)	n.s	−0.57 (0.35)	n.s
Cohabitating	0.77* (0.26)	2.167 [1.301–3.609]	0.41 (0.25)	n.s
Intense psychological violence	—	—	0.70* (0.27)	2.020 [1.200–3.400]
Economic violence	—	—	1.22**(0.34)	3.401 [1.762–6.565]
Sexual violence	—	—	0.79 (0.62)	*n*.s
Nb of previous months with physical violence	—	—	0.13** (0.03)	1.143 [1.089–1.199]
Level 2: Static characteristics (*n* = 84)				
Age	0.00 (0.03)	n.s	0.00 (0.03)	n.s
Education	−0.98** (0.18)	0.377 [0.263–0.541]	−0.79**(0.19)	0.454 [0.313–0.656]
Children	−0.56 (0.47)	n.s	−0.29 (0.54)	n.s
Criminal record	0.30 (0.48)	n.s	0.34 (0.58)	n.s
Partner age	−0.05** (0.01)	0.951 [0.923–0.979]	−0.06**(0.01)	0.944 [0.917–0.971]
Partner daily alcohol use	0.81* (0.45)	2.240 [0.921–5.453]	0.25 (0.58)	n.s
Partner hard drug use	0.30 (0.38)	n.s	0.26 (0.38)	n.s
Partner criminal record	−0.19 (0.37)	n.s	0.05 (0.42)	n.s

*= *p*<0.05; ** = *p*<0.001 (Standard error is in parentheses).

#### Severe Sexual Violence

The results shown in Table 4 highlight the importance of integrating the characteristics of the violence suffered into predictions of the occurrence of severe sexual violence. Although many static factors and several life circumstances appear to be associated with this type of serious violence (Model 3), their effect fades when monthly co-occurrence of other forms of violence is considered. In the end, only partner’s daily alcohol consumption and emotional limitations are associated with severe sexual violence. Those who report daily alcohol consumption by a partner are, on average, at 2.9 times higher risk of experiencing serious sexual violence. The months when women reported having experienced emotional limitations also show, on average, 2.7 times higher risk of serious sexual violence.

**Table 4.** Multilevel logistic regression predicting severe sexual violence.

Severe Sexual Violence	Model 3: Life Circumstances		Model 3: Violence Suffered	
	γ (S.E.)	OR [CI]	γ (S.E.)	OR [CI]
**Level 1: Dynamic characteristics (*n* = 2108)**				
Emotional limitations	0.90* (0.33)	2.464 [1.278–4.748]	0.99** (0.20)	2.698 [1.813–4.016]
Physical limitations	0.45 (0.30)		0.23 (0.32)	
Employment	−0.28* (0.12)	0.758 [0.961–0.599]	−0.22 (0.15)	
In relationship	0.969* (0.41)	2.634 [1.192–5.823]	−0.33 (0.30)	
Cohabitating	0.74** (0.22)	2.101 [1.357–3.253]	0.30 (0.21)	
Intense psychological violence	—	—	0.61** (0.16)	1.843 [1.360–2.496]
Economic violence	—	—	1.73** (0.31)	5.612 [3.081–10.223]
Physical violence	—	—	0.72** (0.21)	2.062 [1.366–3.112]
Nb of previous months with sexual violence			0.07* (0.03)	1.074 [1.006–1.147]
**Level 2: Static characteristics (*n* = 84)**				
Age	−0.01 (0.02)		−0.01 (0.02)	
Education	−0.45* (0.17)	0.641 [0.454–0.905]	−0.11 (0.14)	
Children	0.698 (0.41)		0.52 (0.33)	
Criminal record	−0.49 (0.43)		−0.41 (0.40)	
Partner age	−0.01 (0.01)		−0.00 (0.01)	
Partner daily alcohol use	1.63** (0.35)	5.092 [2.521–10.284]	1.08* (0.36)	2.941 [1.430–6.046]
Partner hard drug use	0.05 (0.33)		−0.07 (0.36)	
Partner criminal record	1.85** (0.47)	6.369 [2.525–16.129]	0.97 (0.38)	

* = *p*<0.05; ** = *p*<0.001 (Standard error is in parentheses).

The monthly occurrence of all forms of violence was associated with severe sexual violence. The risk of experiencing severe sexual violence was 1.8 times higher during months of more intense psychological violence, 5.6 times higher when there was economic violence, and 2.1 times higher when there was physical violence. As with severe physical violence, sexual violence was aggravated over time: each additional month of sexual violence increased the risk of serious sexual violence by 7%.

The results presented show the difficulty of predicting the occurrence of serious physical or sexual violence based on individual characteristics and conventional life circumstances, suggesting that the presence of other kinds of violence is more important in understanding the temporal aspects of acts of severe violence.

## Discussion

The purpose of this study was to describe the context in which serious IPV occurs to determine if it is possible to predict when it is likely to occur. The life history calendar method was used to reconstruct the life trajectories of the women in the sample, making it possible to collect precise information and establish the exact sequencing of events on a monthly basis. Fischer et al. (1989) found that victims and witnesses of crime can provide more detail about their experience when they are asked to reconstruct the context and circumstances surrounding a crime and Hayes (2018) has argued for the benefits of such a method in IPV research. Particular attention was given to the occurrence of different forms of violence and their relationship to serious physical and sexual violence. Using the LHC helped to establish temporal order and incidents of revictimization and made it possible to not only examine monthly variations in different manifestations of IPV in the same trajectory but to identify the factors that influence short-term transitions in the level and kind of serious violence suffered, providing access to details that official data or victimization surveys do not capture. We were also able to collect information about contacting the police as a way of countering IPV. It is important to note that contact with the police is quite rare in the context of severe physical and sexual violence in intimate relationships, which raises questions about the picture of such violence derived from police data: cases of violence recorded by the police appear to represent only the tip of the iceberg of violence and tell us very little about the context surrounding their interventions.

The LHC method may also provide some therapeutic benefit to participants by creating a space in which victims can safely recall painful memories, allowing them to gain some distance from the events and recognize both the dynamics and the sequence of events involved in different forms of violence. LHC makes it possible to tell the story differently—for instance, people who have difficulty describing events may find that writing about them and visualizing them helps them to reveal more. Telling their story may also make them feel heard.

Reconstructing the context of violence requires considering many spheres of life. Such consideration is part of the LHC method and not only facilitates recall (Belli, 1998) but provides access to a multitude of different kinds of explanatory factors. While some studies have shown the benefit of considering different kinds of factors in predicting IPV (e.g., Hayes, 2016; Blondin et al., 2018), studies that take this route are rare. The meta-analysis undertaken by Yakubovich and colleagues (2018) highlights the imbalance in longitudinal research on IPV, with empirical work oriented more toward physical violence even though co-occurrence of different forms of violence is frequently mentioned as the norm (Hamby & Grych, 2012). Our study is distinguished by the simultaneous integration of life circumstances, relationship characteristics, and characteristics of the victim and perpetrator into the models presented, joining the work of those few other researchers (e.g., Frye & Karney, 2006) who have examined the effect of co-occurrence on violence.

Information from LHC showed that IPV for the women in our sample was frequent and diverse. Rates of prevalence and occurrence were high. The vast majority of women interviewed had suffered psychological, physical, and economic violence during the window period and more than a third had experienced sexual violence. A large proportion (52.4%) had experienced more than two forms of IPV out of the four being considered and one or more form of IPV occurred in 80.2% of the months in which women were at risk. These descriptive statistics point to the high level of co-occurrence of IPV in individual trajectories. The diversity of the profiles of violence over time shows not only the uniqueness of individual patterns but also the difficulty of predicting the evolution of this violence based on static characteristics (Dutton et al., 2005; Katerndahl et al., 2010), supporting a dynamic conceptualization of IPV (Capaldi & Kim, 2007).

To better understand the factors and circumstances associated with severe IPV, three categories of factors were integrated into the models: individual characteristics (static factors pertaining to both the victim and his/her partner), life circumstances (dynamic factors such as employment, relationships, and limitations), and the co-occurrence of different forms of violence (dynamic factors). Our results show that victimization pathways are likely to change in the short term and that it is possible to predict changes in the intensity of violence.

Individual characteristics make only a limited contribution to the explanation of serious violence over time. In the two models presented in Table 3, three individual characteristics were found to be significant and help identify trends in the data. Women with higher education and older partners were less at risk of severe physical violence during the window period while women whose partners consumed alcohol daily had an increased risk of serious sexual violence. Only one life circumstance was found to have a significant effect on serious physical or sexual violence: months of cohabitation were related to an increase in the risk of severe physical violence while months in which participant’s emotional limitations were reported were related to increased risk of severe sexual violence.

Different forms of violence suffered in an intimate partner context are of great importance in the prediction of serious violence and provide information about the consequences of co-occurrence. Not only does the integration of co-occurrence diminish the effect of certain individual characteristics and life circumstances but it is also important in predicting severe violence: the presence of other forms of violence in a month exacerbates the risks of serious violence, both sexual and physical. Our analysis, similar to that of Frye and Karney (2006) and Blondin et al. (2018), who report an association between changes in the levels of psychological aggression and the parameters of physical violence, also reveals a link between changes in levels or occurrence of psychological and physical violence as well as a similar association between psychological and sexual violence, indicating the value of considering economic, physical, and sexual violence in predicting the severity of IPV and the close relationship between different forms of violence. This relationship has been reported previously in several works on violence (e.g., Finkelhor et al., 2009; Cuevas et al., 2010) but has received relatively little study in relation to IPV. Our results support Hamby and Grych’s hypothesis that co-occurrence of forms of violence is the norm (at least in the short term) and that it is important to develop a conceptual framework that integrates the kinds of violence that can take place in an intimate relationship, not only to provide a more accurate portrait of the phenomenon but also to guide intervention efforts (Hamby & Grych, 2012).

Another important finding is the aggravating effect of repeated violence. The more violence is repeated, the greater the risk that it will become serious, regardless of other factors. Each additional month in which physical or sexual violence is experienced increases the likelihood that the violence will become more severe, regardless of the characteristics of the victim, the abusive partner, or the relationship. This aggravation over time has been confirmed using police data (Barnham et al., 2017; Kerr et al., 2017; Ouellet et al., 2016) and in qualitative studies that look at the dynamics of violence in an intimate partner context (Winstok, 2008). The results suggest that intervention should be undertaken quickly, to avoid the aggravating effect associated with the accumulation of months of violence.

This study is not without limits. It is important to clarify that, as with other studies using a similar method and time unit (e.g., Horney & al., 1995), the modeling strategy makes it possible to highlight only associations between independent variables and outcome during the same month. We do not pretend to have established a causal chain of events leading to severe violence (nor was this the goal), but instead used a research design focused on association. The result is an exploratory study that recognizes that factors other than the characteristics of victims, aggressors, relationship, and life circumstances considered here may influence behaviors. Although the study focuses on women of different age groups, this research does not take into account several factors related to diversity (e.g., ethnicity, gender, and religion). This is obviously a limit to the scope of our results. In addition, the relationship dynamics examined in this study relate almost exclusively to heterosexual couples in which the victim is a woman, and the aggressor is a man (only one relationship involved a lesbian couple). This finding agrees with that of other research on risk factors, particularly work that falls within the intersectionality framework developed by Kimberle Crenshaw (1992), which suggests that severity of IPV over time could be higher for some women due to their intersecting social positionings. Due to the nature of our sample, these inequalities could not be considered in our models, limiting the scope of the results obtained but encouraging researchers to continue the study using other samples. Although several studies have shown the reliability of data from life history calendars, particularly in order to reconstruct the life experience of victims of intimate partner violence (Hayes, 2018), this method relies on the ability of victims to recall difficult memories. It is therefore possible that in some cases the difficulty of this exercise affected the recall of events. Another limitation relates to the choice of study period and unit of analysis. Episodes of IPV often occur over several years and may be experienced during several relationships. The 3-year observation period does not represent the full trajectory of an individual’s victimization and the unit of time chosen (1 month) may also mask some subtle variations.

Despite these limitations, our results have practical implications. Those who work with victims or abusive partners should be made aware that certain contexts suggest the risk of escalation towards more serious violence and that these contexts can be identified by the diversification and duration of violence. Couples should be made aware that economic and sexual violence are part of a power dynamics that can contribute to the intensification of violence.

The results also have implications for IPV risk assessments. Since the severity of future violence is influenced by recent acts of violence, risk assessment should be based on the short term and focused on the occurrence, diversity, and frequency of different forms of violence. Assessors need to be aware that the kind and severity of violence can change very quickly and that assessments therefore need to be repeated often. Our results suggest that official history of violence is not the best predictor of severe violence, as the criminal record of the spouse loses its association with serious violence when the forms and frequency of IPV are considered. Professionals must therefore assume that the victims with whom they intervene are probably not facing their first experience of victimization, even in the absence of any known contact with the police or the judicial system.

Finally, the results may also have implications for interventions with victims or their violent partners, suggesting that, in keeping with the harm reduction perspective, all interventions that lead to limiting economic and sexual violence and consumption of alcohol or that help violent partners to decrease the frequency of any form of violence are useful in preventing severe violence.

## Conclusion

This study is innovative in that it uses both the LHC method and a multidimensional approach to address the issue of IPV. The perspective adopted is based on the idea that life trajectories, and the changes that occur in them, must be viewed dynamically in relation to past and present experiences. The lack of available data has meant that there are few previous quantitative studies that consider the trajectories of victims of IPV. Like Piquero and colleagues (2006), we conclude that individual victimization trajectories are heterogenous and likely to change in the short term. Co-occurrence of different types of violence was found to be frequent and repeated over time. Our data also reveal a bias in police data on IPV. In our sample, police were contacted in only 3.4% of the months in which violence was recorded. Almost half (41.4%) of those in our sample had never contacted the police, 42.9% had had only one contact, 10.0% two, and 5.7% three. Police data would therefore show that only 15.7% of these women had been victims of repeated violence during the period under study, while 100% had actually been revictimized. Police data thus provide a very partial portrait of reality, which explains, at least in part, the discordant results on the severity of IPV in research based on their data.

The implementation of effective intervention strategies for IPV depends on improving our knowledge and identifying those factors that increase vulnerability as well as those that provide some protection; this study contributes to the development of such knowledge. Understanding the severity of violence requires a complete picture of both the kind and level of violence and the context in which it occurs.

## Notes

1 These data have already been used to understand the evolution of IPV over time (Patard et al., 2020), to examine influences on the dynamics of the frequency of physical violence (Blondin et al., 2018), and to describe the protection strategies adopted (Patard & Ouellet, 2021).

2 In some months (*n* = 53), women were in contact with both a partner and an ex-partner.

3 (21) My partner used a knife or a gun on me, (27) My partner punched or hit me with something that could hurt, (33) My partner choked me, (37) My partner slammed me against a wall, (43) My partner beat me up, (61) My partner burned or scalded me on purpose, (73) My partner kicked me.

4 (19) My partner used force (such as hitting, holding down, or using a weapon) to make me have oral or anal sex, (47) My partner (likes hitting, holding down, or using a weapon) to make me have sex, (57) My partner used threats to make me have oral or anal sex, (75) My partner used threats to make me have sex.
